# Mapping and screening of the tomato *Stemphylium lycopersici* resistance gene, *Sm*, based on bulked segregant analysis in combination with genome resequencing

**DOI:** 10.1186/s12870-017-1215-z

**Published:** 2017-12-29

**Authors:** Huanhuan Yang, Tingting Zhao, Jingbin Jiang, Songbo Wang, Aoxue Wang, Jingfu Li, Xiangyang Xu

**Affiliations:** 1 College of Horticulture, Northeast Agricultural University, Mucai Street 59, Xiangfang District Harbin, 150030 China; 2 BGI, Shenzhen, China

**Keywords:** *S. Lycopersici*, *Sm* gene, Tomato gray leaf spot, BSA, Genome resequencing

## Abstract

**Background:**

Tomato gray leaf spot disease caused by *Stemphylium lycopersici (S. lycopersici)* is considered one of the major diseases of cultivated tomatoes. The only *S. lycopersici* resistance gene, *Sm*, was derived from the wild tomato species *S. pimpinellifolium*. *Sm* has been identified as an effective source of gray leaf spot resistance in tomatoes and has been mapped to tomato chromosome 11. In this study, the first bulked segregant analysis (BSA) combined with genome resequencing for the mapping and screening of the *Sm* candidate gene was performed.

**Results:**

Based on the resequencing results, we identified 50,968 Diff-markers, most of which were distributed on chromosome 11. A total of 37 genes were located in the interval of 0.26-Mb. The gene loci of resistant and susceptible lines were sequenced successfully using PCR products. The relative expression levels of candidate genes in resistant and susceptible lines were confirmed via qRT-PCR, Solyc11g011870.1.1 and Solyc11g011880.1.1 were identified through qRT-PCR. A marker, D5, which was cosegregated with the resistant locus, was identified according to the mutation of the Solyc11g011880.1.1 trait in the resistant line.

**Conclusions:**

The *Sm* gene was mapped to the short arm of chromosome 11. The candidate genes Solyc11g011870.1.1 and Solyc11g011880.1.1 displayed expression patterns related to the resistance response. This study will be valuable for *Sm* cloning and *Sm* gene breeding in tomato.

**Electronic supplementary material:**

The online version of this article (10.1186/s12870-017-1215-z) contains supplementary material, which is available to authorized users.

## Background

Gray leaf spot disease is considered a common, devastating, and damaging disease of plants such as pepper [1], cotton [2], spinach [3] and eggplant [4]. In tomato, it is caused by four species of *Stemphylium*: *Stemphylium solani*, *Stemphylium floridanum* and *Stemphylium lycopersici* [5]. It is considered a major disease of cultivated tomatoes and has threatened tomato-growing areas worldwide [6]. In the early stages, tomato gray leaf spot disease symptoms appear as brownish-black specks that later expand into necrotic lesions with gray centers and dark brown borders. As the disease progresses, the affected leaves become chlorotic and the lesions develop perforated centers, ultimately causing the leaves to become dry and fall off. *S. lycopersici* has been shown to cause tomato gray leaf spot disease based on morphology and molecular identification [7]. However, it is increasingly difficult to control this disease in tomato-growing regions around the world. Therefore, breeding for gray leaf spot resistance will provide an attractive alternative to chemical control.

To date, only the resistance gene *Sm* has been identified as a dominant gene, which was mapped to chromosome 11 in tomatoes [8]. *Sm* was derived from the wild tomato species *S. pimpinellifolium,* which has been used to breed resistant tomato cultivars [9]. The *Sm* gene is considered an effective source of gray leaf spot resistance in tomatoes. To our knowledge, few studies have been conducted on the tomato gray leaf spot resistance gene.

Bulked segregant analysis (BSA) was first proposed by Michelmore et al. [10] and was considered an effective method for identifying markers linked to a target gene [11–13] based on the genotyping of only two bulked DNA samples from groups of individuals with distinct resistant and susceptible phenotypes [14]. Genome resequencing technology, which is based on high-throughput sequencing, is a recently developed high-resolution strategy for single nucleotide polymorphism (SNP), insertion-deletion (InDel) and structural variation (SV) marker discovery and genotyping [15].

Notably, this is the first time that bulked segregant analysis combined with genome resequencing technology has been used for the mapping and screening of candidate genes for the tomato *Stemphylium* resistance gene *Sm*. This study will be valuable for *Sm* cloning and resistance breeding in tomato.

## Methods

### Plant materials and *S. lycopersici* inoculation

Resistant female Motelle (P_1_, kindly provided by the Chinese Academy of Agricultural Sciences), comprising the *Sm* gene, was crossed with the susceptible male Moneymaker (P_2_, kindly provided by the Chinese Academy of Agricultural Sciences). The resulting F_1_ plants were self-crossed to produce F_2_ seeds, and the BC_1_ plants were obtained from a backcross between Motelle and F_1._ lines. These seedlings were bred in a greenhouse under favorable conditions. *S. lycopersici* was plated on potato dextrose agar (PDA) in Petri dishes. The isolated pathogen was incubated at 28 °C for 5–10 days with a 12-h photoperiod. The tomato seedlings were sprayed with a conidial suspension (1 × 10^4^ conidia/ml). The mock-treated plants were sprayed with sterilized water. All seedlings were grown in the greenhouse at 28 °C with a relative humidity >85% [14]. After 3–5 days, symptoms of gray leaf spot disease appeared on the tomatoes.

At the 3–4 leaf stage, all P_1_, P_2_, F_1_, F_2_, and BC_1_ plants were inoculated with *S. lycopersici.* The disease index was evaluated at 7 days post inoculation [16]. The plants were visually assessed for the severity of symptoms on a scale of 1–5 points: 1 point, no symptoms; 2 points, rare lesions; 3 points, few lesions; 4 points, numerous lesions; and 5 points, coalescence of lesions. Plants with a disease index of 0–1 were regarded as resistant, and those with a disease index ≥2 were regarded as susceptible.

After inoculation, the F_2_ plants were used for genetic analysis and bulked segregate analysis (BSA). The parents (P_1_ and P_2_) and the F_2_ lines were prepared for genome resequencing and the detection of molecular markers, respectively. The resistant pool (F_2_R-pool, 25 resistant plants) and the susceptible pool (F_2_S-pool, 25 susceptible plants) were built by screening resistant and susceptible plants from the F_2_ populations [17]. The CTAB method was used for DNA extraction from young leaves, including the parents and the F_2_ lines. Bulked DNA samples were also subjected to the CTAB method by mixing equal amounts of DNA at a final concentration of 200 mg [18].

### Resequencing and association analysis

The genomic DNA of each individual plant was extracted and then fragmented randomly. Adapter ligation and DNA cluster preparation were then performed, followed by Hiseq2000 sequencing. SOAP2 software (http://soap.genomics.org.cn/soapaligner.html) was used for sequencing reads, which were mapped to the reference genome sequence [19]. The sequencing depth and coverage compared with the reference genome were calculated based on the alignments. SNPs and InDels in the sequenced genome were detected using SOAP snp (http://soap.genomics.org.cn/soapsnp.html) and SOAP indel, respectively. SNP annotation (including synonymous and non-synonymous SNP mutations) and InDel annotation were performed according to the tomato reference genome (estimated size of 0.9 Gb) [20].

In this study, an average of at least 20× were generated from each sample to detect all SNPs and lx for each progeny (25 for each pool) [15]. Raw sequence reads (150 bp in length) were filtered and trimmed for quality control and adaptor removal [15]. A library was constructed for each sample, and all clean Q20 (%) values for the four libraries (P_1_, P_2_, F_2_R-pool and F_2_S-pool) were greater than 96%, indicating that the data quality was very high. Primary sequencing data were cleaned by removing reads with adapters, and a low-quality read was defined based on the number of low-quality reads (<20). Low-quality reads were removed, and the remaining high-quality data were used for mapping [21]. An SNP was defined according to its presence in the two parent lines and markers with a depth no less than 5 and a base quality >20. A homozygous and variant SNP marker, which originates from two parent lines, was used for further analysis [22, 23].

The resistant phenotype is dominant, and the ratios of resistant plants should be consistent with those of the susceptible plants for the unrelated markers [24]. Therefore, only the genotype of the P_2_ plants could be found in terms of related markers. The SNP index is a method of marker association analysis, which is used to judge differences in genotype frequencies between pooled samples [25]. The SNP indexes of the resistant and susceptible pools were 0.25 and 0.75, respectively, and those SNPs were used to analyze the associated regions of candidate genes [24]. The resequencing and association analysis were performed by BGI Tech (Shenzhen, China).

### Parental genome sequencing and candidate gene screening

The alignment results were used to calculate the average sequencing depth and coverage [26, 27]. All SNP and InDel polymorphisms were detected using the mapped reads. In this part of the study, DNA from the parents was used for sequencing, read mapping, and analysis of SNPs and InDels [28]. The Heinz 1706 tomato [20] was used as the reference genome for read mapping. All the SNPs in the association region were used for BSA combined with genome resequencing. Genes in the association region were annotated based on the NCBI and SGN (http://solgenomics.net/) websites. SNPs that were consistent with the susceptible genotype in the F_2_S-pool were used to narrow the candidate region.

### Quantitative real-time PCR analysis of candidate genes

Candidate gene expression analysis was performed using qRT-PCR. P_1_ and P_2_ were inoculated at the 3–4 true leaf stage. Young leaves were collected at 0, 3, 5 and 8 days after inoculation. Total RNA was extracted using the TRIzol reagent method, with three biological repeats. Reverse transcription was performed using the TaKaRa M-MLV Reverse Transcriptase (RNase H-) reverse transcription kit according to the operating instructions.

The qRT-PCR mixture contained 10 μL of 2xTrans Start Top Green q PCR Super Mix (Trans Gen, China), 1 μL of each primer, 2 μL of the cDNA template (1:5 dilution) and the appropriate volume of sterile distilled water for a total volume of 20 μL. The thermal conditions were as follows: 95 °C for 10 min, followed by 40 cycles of 95 °C for 5 s, 59 °C for 15 s, and 72 °C for 30 s. To detect primer dimerization or other artifacts of amplification, a melting-curve analysis was performed immediately after the completion of qRT-PCR (95 °C for 15 s, 55 °C 15 s, then slowly increasing the temperature by 0.5 °C per cycle to 95 °C with continuous measurements of fluorescence). The data analysis was performed using the 2^–∆∆CT^ method (Livak and Schmittgen, 2001) with EFa1 (R: 5’-CCACCAATCTTGTACACATCC-3’ S: 5’-AGACCACCAAGTACTACTGCAC-3′) as a reference gene for normalization.

### Sequencing of candidate gene loci and DNA sequence analysis

Primer 5.0 software was used to produce the sequences of candidate genes, and the primers of candidate genes are shown in Table 4. Reference genome sequences of candidate genes originated from the SGN.

A PCR purification kit (Takara) was used to purify parental PCR products. These purified products were then cloned into a pMD18-T vector (Takara) for sequencing. All fragments that came from sequencing were uploaded to GenBank.

### Marker development and linkage analysis

To develop more polymorphic markers between Motelle and Moneymaker based on sequence variations in or near the resistant gene trait, the sequences of candidate genes were obtained from the SGN, and Primer 5.0 software was used to develop the marker primers. After PCR and sequencing, eight markers, including CAPS markers and SCAR markers, were developed to screen an F2 population of 519 plants for linkage analysis.

## Results

### Identification of the pathogen and genetic analysis of the *Sm* gene

**Fig. 1:**
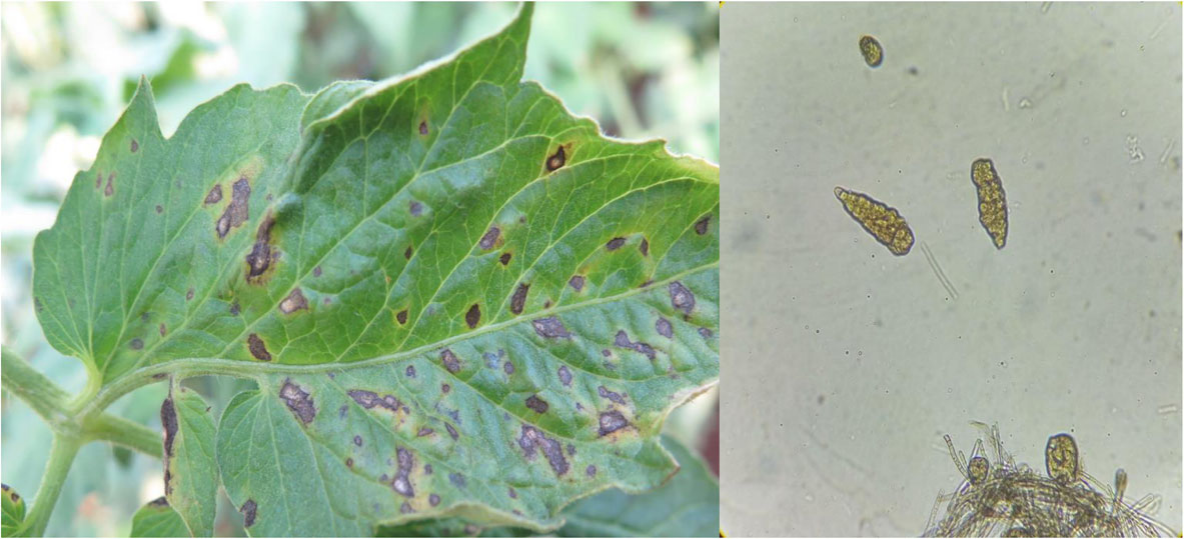
**a** Symptoms of gray leaf spot disease on tomato leaves; (**b**) Conidia and conidiophores of *Stemphylium lycopersici* as seen under 400X magnification

In this study, genomic DNA of the pathogen was extracted, and the internal transcribed spacer (ITS) regions were amplified and sequenced with the primers ITS1 and ITS4. All sequences were submitted to GenBank (accession nos. KX858848, KX858849). BLAST search results revealed that all sequences exhibited 99% identity with *S. lycopersici* [29]. Figure 1a shows the symptoms of gray leaf spot disease on tomato leaves. Figure 1b shows the conidia and conidiophores of *S. lycopersici* as observed under 400X magnification.

Finally, we confirmed that gray leaf spot disease was caused by *S. lycopersici* based on morphological characteristics and molecular identification. Motelle and F_1_ plants were resistant to *S. lycopersici*, while Moneymaker plants were susceptible. The segregation ratio between resistant and susceptible plants in the F_2_ population was 3:1. The segregation ratio between resistant and susceptible plants in BC_1_ plants was 1:1 (Table 1).

**Table 1:** Genetic analysis of *Sm* disease-resistance in different generations

		No. of plants		Expected segregation	
Generation	Total	Resistant (R)	Susceptible (S)	ratio R:S	χ2
P_1_ (Motelle)	80	80	0		
P_2_ (Moneymaker)	80	0	80		
F_1_	50	50	0		
F_2_	519	379	140	1:3	0.93
BC_1_P_1_	185	89	96	1:1	0.20

P_1_: Resistant female, Motelle; P_2_: Susceptible male, Moneymaker. The F_1_ plants were self-crossed to produce F_2_ seeds, and the BC_1_ plants were obtained from a backcross between P_1_ and F_1_ lines

### Parental genome sequencing and candidate gene screening

In this study, a total of 67 Gb of data, including 447 M reads, and 89 Gb of data, including 593 M reads, were obtained through parental genome resequencing and F_2_ bulked segregant analysis, respectively (Table 2).

**Table 2:** Summary of the sequencing data

Sample	GC_rate (%)	Q20_rate (%)	Q30_rate (%)	Bases (G)	Average depth of sequencing	Genome coverage_rate (%)	Unique_map_read_rate (%)	Map_read_rate (%)
P2	39.29	96.68	92.66	33.68	40.87	88.61	78.34	81.81
P1	38.92	96.35	92.06	33.38	40.51	88.5	79.77	83.63
F2S	38.93	96.42	92.13	46.05	55.89	88.89	78.29	81.94
F2R	38.65	96.56	92.43	42.98	52.17	88.76	79.9	83.62

Average depth of sequencing = total bases/genome size; Genome coverage_rate = mapped genome bases / total genome size; Unique_map_read_rate: the rate of reads in only one place is aligned on the genome; Map_reads_rate: the rate of reads is aligned on the genome

**Fig. 2:**
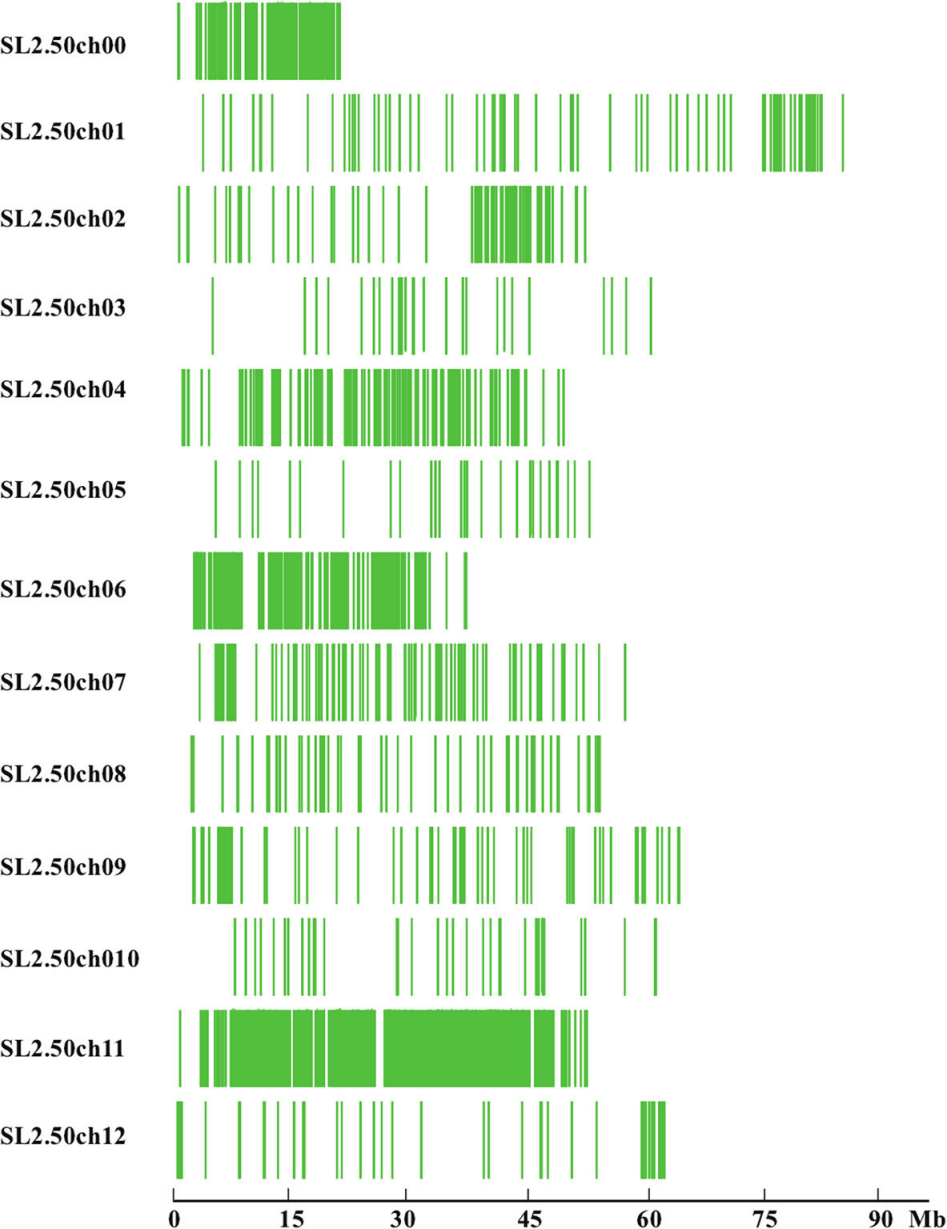
Marker distribution on the chromosomes

Based on the resequencing results, 50,968 Diff-markers in parent lines were obtained, and 46,941 of these markers were distributed on chromosome 11. A distribution diagram of polymorphic markers (green lines) on 12 chromosomes was drawn according to the results of the resequencing positioning on the genome (Fig. 2). A region was considered an association region based on three or more consecutive Diff-markers. We found that 37 genes were distributed in the association region on chromosome 11 (Table 3). SNPs that were consistent with the susceptible genotype in the F_2_S-pool were used to narrow the candidate region.

**Table 3:** Statistics of the candidate region

Start position	End position	Region Size (Mb)	Gene number
4,570,194	4,830,659	0.26	37

**Fig. 3:**
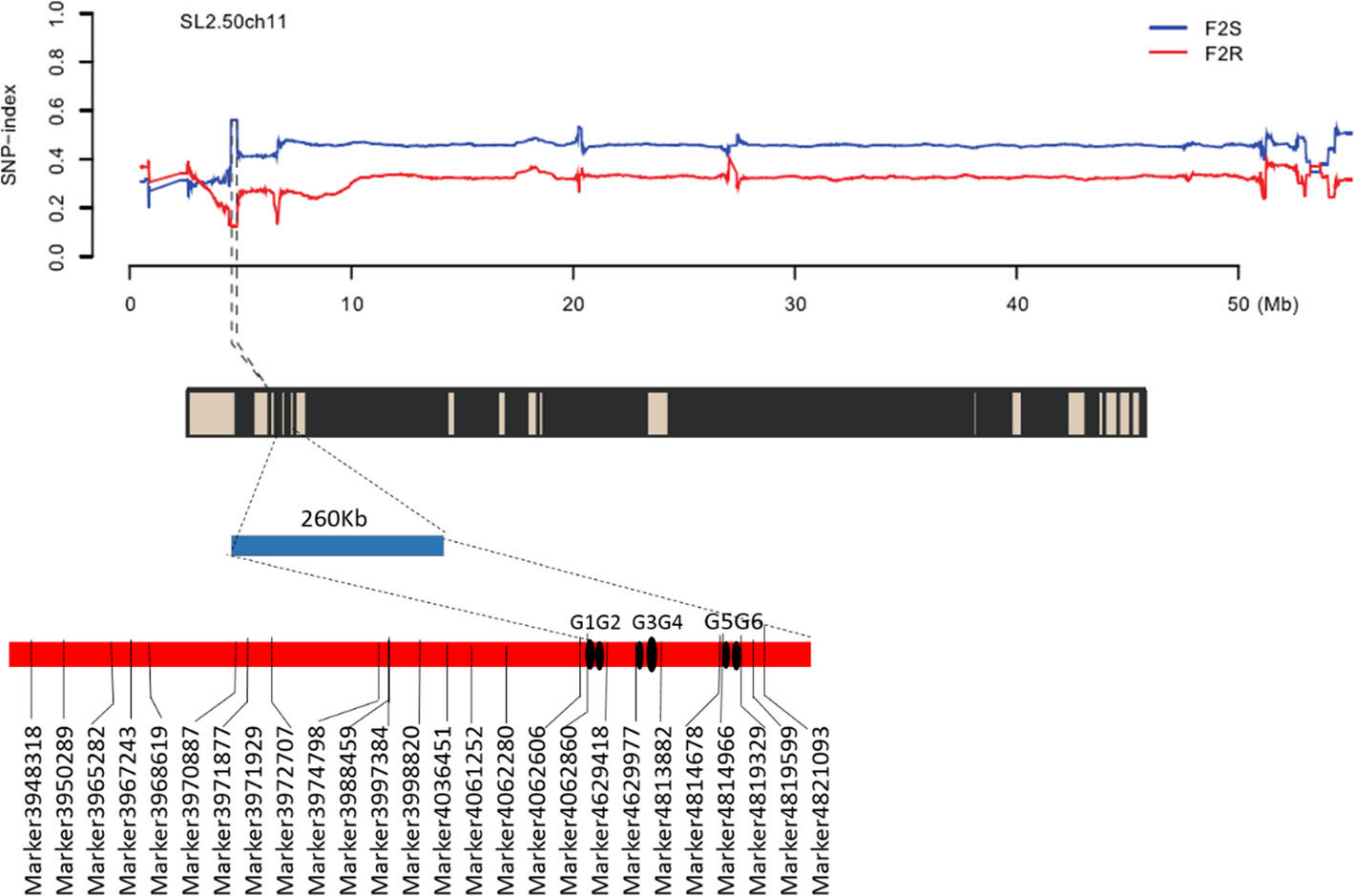
Genetic and physical maps of mapping regions and the mapping analysis process. The association region was 260 kb in size. Eight markers were distributed in the region and six candidate genes (G1-G6) in a 260-kb region were screened out based on the F_2_ BSA analysis and parental resequencing. G1, Solyc11g011520.1.1; G2, Solyc11g011530.1.1; G3, Solyc11g011690.1.1; G4, Solyc11g011750.1.1; G5, Solyc11g011870.1.1; G6, Solyc11g011880.1.1

To further narrow the candidate region, SNPs in the association region were screened by resequencing combined with the F_2_ BSA data and were analyzed. All SNPs, including 8 with an SNP index close to 0.33, were located in the interval of 0.26-Mb region (Table 4) (Additional file 1: Table S1). Based on the results of the association analysis and gene function annotation, six genes were screened out: Solyc11g011520.1.1, Solyc11g011530.1.1, Solyc11g011690.1.1, Solyc11g011750.1.1, Solyc11g011870.1.1 and Solyc11g011880.1.1 (Fig. 3).

**Table 4:** Primers used for qRT-PCR analysis and candidate loci sequencing

Primer name	Forward primer sequence (5′-3′)	Reverse primer sequence (5′-3′)
qRT-PCR primers		
qRT-520	GAACTCCGCCTGAGATGCAA	TTGTGCTTTAGGCGATGCAC
qRT-530	GACTCCAACTCGACACTCGT	TCGATCTCGGGACAACAAGG
qRT-690	AAGAATTTGGGCTGCCGAGT	TGGATTCCACTTTCTTGGTGC
qRT-750	AGGCCATGGGGAAAGTATGC	GCGATTCGCGCACTTTATCT
qRT-870	TTGGACGCATGCTTTGAACC	CCGCATGTTTAGGCGAACAG
qRT-880	GATTCGCCACGACACAACAG	AAGAGTTGTAGTGCCACGGG
EFα1	CCACCAATCTTGTACACATCC	AGACCACCAAGTACTACTGCAC
Sequencing primers		
880–1	CCCGTGGCACTACAACTCTT	TCTGCTTTCGCTCTGCTTGA
880–2	CGAATGCTTTAAGCCCCGTG	CCGGATGCTTCCCTCTTTGT
870–1	TTGGACGCATGCTTTGAACC	CCGCATGTTTAGGCGAACAG
870–2	TGTTCGCCTAAACATGCGGA	CCCAAGTTCCATCAAGGCCA

### Quantitative real-time PCR analysis

**Fig. 4:**
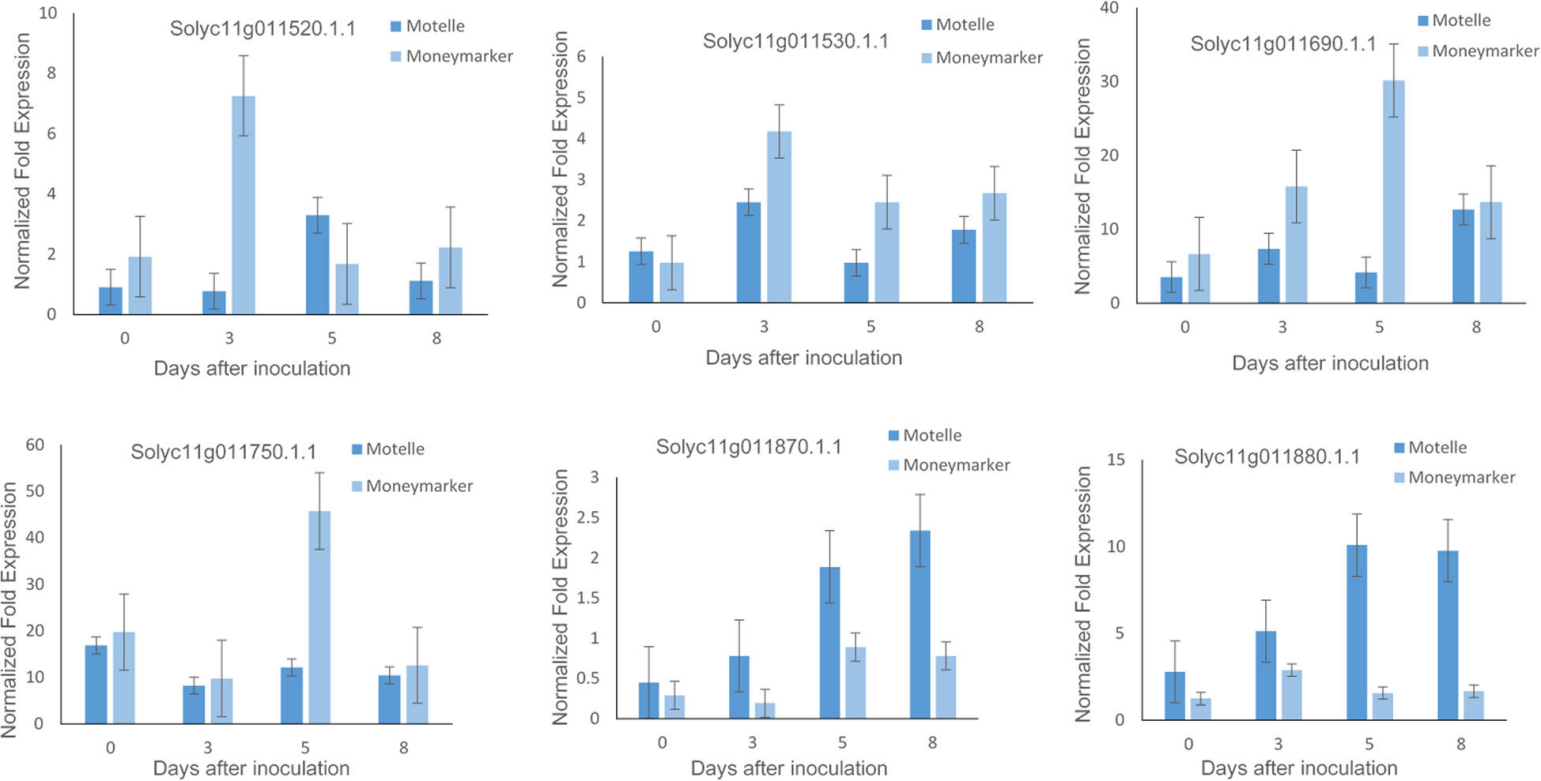
qRT-PCR expression analysis of the six candidate genes in the susceptible (Moneymaker) and resistant (Motelle) lines

The relative expression levels of candidate genes in Motelle and Moneymaker were confirmed using qRT-PCR. The results showed that Solyc11g011870.1.1 and Solyc11g011880.1.1 presented expression patterns related to the resistance response (Fig. 4). The primer sequences for all candidate genes are reported in Table 4. These two candidate genes were expressed at a low level before inoculation, which increased slightly after inoculation. Their expression levels then increased rapidly 5 days after inoculation and continued to increase during the following days. In particular, compared with 0 days, the expression levels of the two resistance-related genes increased at 5 and 8 days after inoculation. Nevertheless, the expression levels of *the other four genes* were incompatible with a relationship with resistance. In conclusion, the qRT-PCR results indicated that the expression levels of Solyc11g011870.1.1 and Solyc11g011880.1.1 were compatible with a relationship with resistance. The other four genes all showed expression patterns unrelated to the resistance response.

In a previous study, we performed a transcriptome analysis of the *Sm*-mediated resistance response to *S. lycopersici* in tomatoes. Our RNA-Seq results showed that Solyc11g011870.1.1 and Solyc11g011880.1.1 were specifically up-regulated (Log2Fold-Change ≥2) in the Motelle line after 5 days of inoculation compared with the Moneymaker line. Therefore, the expression profiles of Solyc11g011870.1.1 and Solyc11g011880.1.1 were verified in our RNA-Seq analysis. The clean data from all samples have been submitted to the NCBI (http://www.ncbi.nlm.nih.gov/biosample) under accession number SRP097450.

### Candidate gene sequencing and sequence analysis

The Solyc11g011870.1.1 and Solyc11g011880.1.1 loci of Motelle and Moneymaker were successfully sequenced using the PCR products (GenBank: MF059094, MF059095, MF059096, MF059097). All other association region gene loci of Motelle and Moneymaker were also sequenced successfully using the PCR products. Sequence alignment showed that the DNA sequences of thirty-two gene loci contained SNPs between Motelle and Moneymaker. There were several SNPs located in coding regions at some candidate gene loci, but no difference in the distribution of conserved domains was found between Motelle and Moneymaker.

**Fig. 5:**
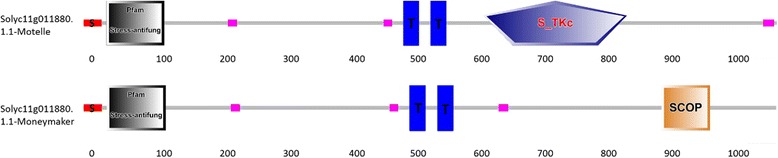
The protein structure of the candidate loci in Motelle and Moneymaker. The Solyc11g011880.1.1 conserved protein structures in Motelle and Moneymaker were different; Solyc11g011880.1.1-Moneymaker included a signal peptide, a Stress-antifung, two transmembrane regions and an SCOP domain, and Solyc11g011880.1.1-Motelle included a signal peptide, a Stress-antifung, two transmembrane regions and an S_TKc domain. The “S” inside shows the signal peptide domain and “T” shows the transmembrane region

Interestingly, at the Solyc11g011880.1.1 locus, a difference in the domain distribution was detected between Motelle and Moneymaker. In the Motelle genotype, there was a 56-bp insertion in the CDS region compared with the Moneymaker genotype, and this insertion changed the ORF. Based on the analysis of the conserved domain and its characteristics, this insertion in the coding region alters the conserved SCOP domain to S_TKc (Fig. 5). The conserved protein structure of Solyc11g011880.1.1 from Moneymaker included a signal peptide, a Stress-antifung domain, two transmembrane regions and an SCOP domain. The conserved protein structure of Solyc11g011880.1.1from Motelle in the CDS region included a signal peptide, a Stress-antifung domain, two transmembrane regions and an S_TKc domain.

### PCR validation and marker development

**Fig. 6:**
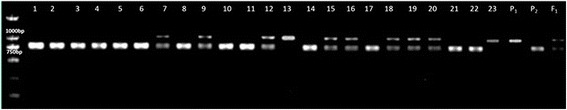
Marker D5 amplification in different generations. P_1_: Motelle; P_2_: Moneymaker; F_1_: Motelle × Moneymaker; 1–23: F_2_ individuals

The SCAR marker D5 (forward primer: 5′- CCCGTGGCACTACAACTCTT-3′; reverse primer: 5′- TCTGCTTTCGCTCTGCTTGA-3′) was cosegregated with the resistance locus. The D5 was designed according to a 56-bp insertion in the resistance trait of Motelle. An 876-bp sequence was amplified from resistant plants, while an 820-bp sequence was amplified from susceptible plants, and the two sequences were amplified in the F_1_ plants (Fig. 6). Among the total F_2_ populations, the susceptible samples displayed the Moneymaker genotype, resistant samples displayed the Motelle or F_1_ genotype, and five susceptible plants showed the Motelle genotype. The results revealed that the segregation ratio of the Solyc11g011880.1.1 genotype between resistant (876 bp) and susceptible (820 bp) individuals of the F_2_ population was 3:1. The phenotype identification and molecular marker identification results for all F_2_-line plants were nearly consistent.

## Discussion

### The *Sm* gene maps to chromosome 11

The *Sm* gene was previously assigned to the long arm of chromosome 11 between TG110 and T10 in 1991. In this study, the *Sm* gene was mapped to the short arm of chromosome 11, and one association region of the *Sm* gene was identified based on the results of F_2_ bulked segregant analysis in combination with genome resequencing technology. Although some Diff-markers were found on the long arm of chromosome 11, they did not form association regions in the association analysis. To the best of our knowledge, few studies of this gene have been performed in recent years.

### The 56-bp insertion of Solyc11g011880.1.1 in Motelle in the coding region changes the conserved domain

Pathogens harbor toxic and avirulent (AVR) genes, and host plants exhibit resistance (R) and susceptible (S) genes. A plant can develop induced resistance only when a pathogen carrying an avirulent gene infects a host plant carrying a corresponding R gene; otherwise, the plant will be infected [30]. However, it is also theoretically suggested that there is a potential for resistance in plants carrying either R or S genes. The specialization between plants and pathogens is based on the level of receptor recognition. Therefore, all plant R genes encode receptor proteins. Thus, whether induced disease resistance occurs depends on the properties of mutual plant-for-pathogen and gene-for-gene recognition. Plant disease resistance is generally defined by the gene-for-gene hypothesis [31], which states that when an avirulent (AVR) gene product of a pathogen is specifically recognized, an R gene encoding a receptor protein induces the process of plant resistance.

Stress-antifung proteins are considered effective in resistance to fungal diseases. Ginkbilobin-2 (Gnk2) is an antifungal protein, which was identified in the endosperm of Ginkgo seeds, that was found to play a vital role in the development of phytopathogenic fungi (e. g., *Fusarium oxysporum)*. Previous studies indicated that Gnk2 was very similar to the extracellular domain of cysteine-rich receptor-like kinases (CRK) in *Arabidopsis*. These findings also demonstrated that CRKs could be induced by pathogen infection, a series of responses to reactive oxygen species or salicylic acid as a component of hypersensitivity reactions, which is a typical system of programmed cell death [32]. A similar result was obtained in the present study, in which the sequence of Solyc11g011880.1.1 was found to be identical to the extracellular domain of cysteine-rich receptor-like kinases from *Nicotiana tomentosiformis*. Therefore, Solyc11g011880.1.1 is a CRK that may be involved in hypersensitivity reactions. According to the reference genome, the functions of the candidate gene Solyc11g011880.1.1 were annotated as follows: receptor-like kinase (RLK), receptor-like protein (RLP) and putative resistance protein (PRR) with an antifungal domain. Interestingly, at the Solyc11g011880.1.1 locus, a difference in the domain distribution was detected between Motelle and Moneymaker. In the Motelle genotype, there was a 56-bp insertion in the fifth exon of the CDS region compared with the Moneymaker genotype, and this insertion altered the ORF. Based on the analysis of conserved domains and characteristics, this insertion in the coding region changes the SCOP conserved domain to S_TKc. STKs catalyze the transfer of the gamma-phosphoryl group from ATP to serine/threonine residues on protein substrates. The subfamily of IRAKs includes plant RLKs, and the RLKs include *Arabidopsis thaliana* BAK1. Research has shown that BAK1 functions in pathways involved in plant resistance to pathogen infection [33].

Our results indicated that conserved domain changes in Solyc11g011880.1.1 of Motelle may result in an R gene encoding a receptor protein, causing the avirulent (AVR) gene products of a pathogen to be specifically recognized. Furthermore, the entire process of plant disease resistance is probably induced by the R gene Solyc11g011880.1.1 of Motelle, indicating that the Solyc11g011880.1.1 candidate gene of Motelle may be the target gene, *Sm*. Transcriptome analysis of the *Sm*-mediated resistance response to *S. lycopersici* in tomatoes was carried out within the scope of our research. The RNA-Seq results showed that Solyc11g011870.1.1 and Solyc11g011880.1.1 were specifically up-regulated (Log_2_Fold-Change ≥2) in the Motelle line after inoculating the plants for 5 days compared with the Moneymaker line.

In conclusion, according to the RNA-Seq results and the bulked segregant analysis performed in combination with genome resequencing technology, the candidate gene Solyc11g011880.1.1 in the Motelle line may be our target gene, *Sm*. As two candidate genes, Solyc11g011870.1.1 and Solyc11g011880.1.1 were found in this target region, and the function of these two genes will be verified through virus-induced gene silencing (VIGS). Functional verification of the candidate genes in the resistant tomato line Motelle is currently ongoing in our laboratory.

### The marker D5 can be used in marker-assisted selection (MAS) breeding

The marker D5 was cosegregated with the resistance locus. Interestingly, an 876-bp sequence was amplified in resistant plants, while an 820-bp sequence was amplified in susceptible plants, and the two sequences were amplified in the F_1_ plants. This marker was tested in F_2_ individuals. However, five F_2_ individuals showed an inconsistent genotype. Considering the possible explanations for this finding, we assumed that the resistance was controlled by an incomplete dominant gene.

Although several plants were inconsistent with D5 tests, verification of the D5 marker during genotype identification is required for tomato MAS breeding. The results of the research will provide a basis for future MAS breeding and studies on the mechanism of tomato gray leaf spot disease resistance.

## Conclusions

In this study, the F_2_ bulked segregant analysis combined with genome resequencing were used to locate the *Sm* gene. A total of 50,968 Diff-markers were obtained, most of which were distributed on chromosome 11. A 0.26-Mb region with 37 genes was obtained the resequencing results. The candidate genes Solyc11g011870.1.1 and Solyc11g011880.1.1 were identified through qRT-PCR, which showed related expression patterns. This study will provide a basis for *Sm* cloning and application of the *Sm* gene in breeding.

## Additional file

Additional file 1: Table S1. Results from bulked segregant analysis in combination with genome resequencing. (DOCX 15 kb)

## Abbreviations

Avr: Avlrulence gene
BSA: Bulked segregate analysis
CAPS: Cleaved amplified polymorphic sequences
CTAB: Cetyl trimethyl ammonium bromide
HR: Hypersensitive response
InDel: Insertion/deletion
MAS: Marker assistant selection
NCBI: National center for biotechnology information
ORF: Open reading frame finder
PRR: Putative resistance protein.
qRT-PCR: Quantitative real-time PCR
R gene: Resistance gene
RLK: Receptor-like kinase
RLP: Receptor-like protein
*S. lycopersici*: *Stemphylium lycopersici*
SCAR: Sequence characterized amplified region
SGN: SOL Genomics network
SNP: Single nucleotide polymorphism
VIGS: Virus-induced gene silencing
