# Supplementary material for: Mapping and screening of the tomato Stemphylium lycopersici resistance gene, Sm, based on bulked segregant analysis in combination with genome resequencing
Source: BMC Plant Biol. 2017 Dec 29;17:266. doi: 10.1186/s12870-017-1215-z (PMC5747103; doi:10.1186/s12870-017-1215-z)
Supplement: Additional file 1: Table S1. — Results from bulked segregant analysis in combination with genome resequencing. (DOCX 15 kb) [file 12870_2017_1215_MOESM1_ESM.docx]

**Table S1 Results from bulked segregant analysis in combination with genome resequencing**

Diff-Marker name genome positon Motelle Moneymaker F2R pool F2S pool

| Marker3950289 | 3950289 | T | G | T/G | G |
| --- | --- | --- | --- | --- | --- |
| Marker3965282 | 3965282 | A | G | A/G | G |
| Marker3967243 | 3967243 | C | T | C/T | T |
| Marker3968619 | 3968619 | T | C | T/C | C |
| Marker3970887 | 3970887 | T | C | T/C | C |
| Marker3971877 | 3971877 | T | A | T/A | A |
| Marker3971929 | 3971929 | G | T | G/T | T |
| Marker3972707 | 3972707 | A | G | A/G | G |
| Marker3974798 | 3974798 | T | C | T/C | C |
| Marker3988459 | 3988459 | G | A | G/G | A |
| Marker3997384 | 3997384 | G | A | T/G | A |
| Marker3998820 | 3998820 | T | G | A/T | G |
| Marker4036451 | 4036451 | A | T | A/T | T |
| Marker4061252 | 4061252 | T | C | T/C | C |
| Marker4062280 | 4062280 | G | A | G/A | A |
| Marker4062606 | 4062606 | C | A | C/A | A |
| Marker4062860 | 4062860 | G | A | G/A | A |
| Marker4629418 | 4629418 | A | T | A/T | T |
| Marker4629977 | 4629977 | C | T | C/T | T |
| Marker4813882 | 4813882 | G | A | G/A | A |
| Marker4814678 | 4814678 | A | G | A/G | G |
| Marker4814966 | 4814966 | G | A | G/A | A |
| Marker4819329 | 4819329 | C | G | C/G | G |
| Marker4819599 | 4819599 | T | A | T/A | A |
| Marker4821093 | 4821093 | A | G | A/G | G |
